# Mapping 35 years of medical specialty theses on clinical nutrition in Türkiye: the rise of the muscle-focused paradigm

**DOI:** 10.1590/1806-9282.20260075

**Published:** 2026-07-31

**Authors:** Zeynep Irmak Kaya

**Affiliations:** 1 Eskişehir City Hospital, Department of Internal Medicine and Nutrition – Eskişehir, Türkiye.

**Keywords:** Nutritional sciences, Sarcopenia, Malnutrition, Diagnostic imaging, Body composition, Geriatrics

## Abstract

**OBJECTIVE:**

The aim of this study was to analyze the thematic and methodological evolution, alignment with global literature, and publication conversion rates of medical specialty theses in clinical nutrition in Turkey between 1990 and 2025.

**METHODS:**

A total of 750 theses were retrospectively analyzed from the Turkish National Thesis Center database. Data were evaluated for thematic content, diagnostic tools, and interdisciplinary distribution using Callon’s centrality/density metrics and segmented regression models.

**RESULTS:**

The dominant research themes were sarcopenia and muscle health (36.5%), nutritional support (28.0%), and malnutrition scores (26.4%). A strong thematic association was found between sarcopenia and frailty, primarily concentrated in geriatrics. Geriatrics exhibited the highest statistical density for sarcopenia and frailty themes (Adjusted Residual: +6.1). Objective muscle measurements were included in 48.2% of theses, with 65% of these utilizing computed tomography-based “opportunistic imaging.” Segmented regression identified 2015 as a significant structural breakpoint in thesis production (p<0.001). The overall publication rate was 28.4%, with the highest conversion observed in sarcopenia-focused theses (42.0%; 95%CI 36.2–47.8). Conversely, cachexia was the least represented area (1.2%).

**CONCLUSION:**

Clinical nutrition research in Türkiye has evolved into a muscle-focused, phenotype-based paradigm, with geriatrics serving as the primary driver. While sarcopenia has become central to clinical research, complex wasting syndromes like cachexia remain academically neglected. These findings demonstrate that geriatric nutrition plays a decisive role in national academic output, though more holistic and multidisciplinary research strategies are needed for future academic training.

## INTRODUCTION

Over the past three decades, clinical nutrition has evolved from a supportive measure into a proactive and integral component of modern medical treatment. This transformation underscores that nutrition is no longer merely a modifier of disease course, but a direct determinant of morbidity, mortality, functional capacity, and survival^1,2^. Disease-related malnutrition remains highly prevalent among hospitalized patients and is associated with poorer clinical outcomes as well as a substantial economic burden on healthcare systems^3,4^.

In parallel, nutritional assessment has undergone a major paradigm shift. Traditional reliance on isolated biochemical markers has been replaced by multidimensional approaches that integrate functional and morphological parameters. The Global Leadership Initiative on Malnutrition (GLIM) criteria^1^ and the European Working Group on Sarcopenia in Older People 2 (EWGSOP2) consensus^5^ exemplify this evolution by standardizing diagnostic frameworks and encouraging the use of advanced imaging and functional assessment tools in clinical practice.

Despite these global advances, the implementation of clinical nutrition research and practice varies considerably between countries, influenced by differences in academic culture, specialist training structures, and national research priorities. In Turkey, scientific production in medicine is largely driven by specialist and subspecialty theses, which generate original, practice-based data and hold substantial potential to contribute to the national and international literature^6,7^.

However, this potential has not been fully realized, as only a limited proportion of theses are converted into publications in internationally indexed journals. This gap highlights the need for a systematic evaluation of thesis-based research and for strategies aimed at strengthening academic productivity during specialist training^8,9^.

Bibliometric analysis provides a robust and objective framework for identifying publication trends, dominant research themes, and knowledge gaps within a scientific field^10^. Accordingly, this study presents the first comprehensive bibliometric analysis of medical specialty and subspecialty theses on clinical nutrition registered in the Turkish National Thesis Center between 1990 and 2025. By examining temporal trends, thematic distributions, and interdisciplinary interactions, the study aims to assess the alignment of national academic output with the global literature and to support the development of more data-driven and effective research strategies in clinical nutrition.

## METHODS

### Study design and ethical considerations

This descriptive, retrospective bibliometric study aimed to characterize medical specialty and subspecialty theses in the field of clinical nutrition in Turkey. The methodology was based on established bibliometric principles and incorporated performance analysis and science mapping approaches^10^.

All data were obtained from the publicly accessible and anonymized database of the Higher Education Council (YÖK) National Thesis Center. As no individual-level or patient-identifiable data were used, ethical committee approval was not required.

### Data source and search strategy

The search was conducted on December 15, 2025, covering all theses registered between January 1, 1990, and December 15, 2025, corresponding to the period in which clinical nutrition-related theses first appeared in Turkey.

Titles, abstracts, and keywords were screened using Boolean operators (AND/OR/NOT). Core search terms included sarcopenia, cachexia, frailty, enteral and parenteral nutrition, malnutrition, nutritional risk, bioimpedance, muscle ultrasonography, and body composition, along with related terms.

### Eligibility criteria

Theses were included if they originated from medical specialties or subspecialties, addressed clinical nutrition, malnutrition, or muscle health as a primary focus, and had fulltext availability.

Non-medical theses, duplicate records, animal or basic science studies without clinical relevance, and records with restricted or missing full text were excluded. Of 855 screened theses, 750 met the inclusion criteria, reflecting clinically oriented medical research.

### Data extraction and reliability

Data were manually extracted using a standardized, pilot-tested Excel template. Each thesis was independently reviewed by two researchers, and key variables—including year, institution, specialty, thematic focus, assessment tools (e.g., GLIM, EWGSOP), study design, and publication status—were recorded.

To assess reliability, 20% of the theses were re-evaluated by a third researcher, yielding high inter-rater agreement (Cohen’s κ=0.92).

### Bibliometric and thematic analysis

The bibliometric evaluation combines performance analysis with thematic science mapping. The temporal trends were the subject of assessment using annual production data and segmented regression models. The purpose of this was to identify structural breakpoints in research output. Owing to the intrinsic heterogeneity inherent in keyword indexing within the national database, and the marked predominance of Turkish-language abstracts, thematic classification was conducted via expert-guided manual coding. This qualitative approach was prioritized to ensure precise disambiguation of clinical terminology and to maintain high thematic integrity across a 35-year longitudinal dataset. Thematic positioning was operationalized using Callon’s density and centrality metrics, derived from a manually constructed co-occurrence matrix of the validated research themes^11^. Centrality was calculated as the sum of external links between a specific thematic node and other themes, thus representing the theme’s relative prominence and structural importance within the national research network. Density was calculated as the strength of internal links within a thematic cluster, reflecting its conceptual maturity and internal development. In order to ensure scientific rigor, these metrics were normalized and mapped onto a two-dimensional strategic diagram (centrality on the x-axis, density on the y-axis), thus allowing for the classification of research domains into motor, niche, emerging, or transversal themes.

### Statistical analysis

Analyses were performed using International Business Machines Statistical Package for the Social Sciences (IBM SPSS) Statistics version 26.0 (IBM Corp., Armonk, NY, USA)^12^. Data were summarized using frequencies and percentages. Pearson’s chisquare test was used for categorical comparisons.

Segmented regression analysis identified changes in thesis production trends, with breakpoints assessed using Davies’ test and model fit evaluated by adjusted R^2^ and Akaike Information Criterion. Statistical significance was set at p<0.05.

### Publication conversion analysis

Thesis-to-publication conversion was assessed through systematic searches in Google Scholar, PubMed, and TR-Dizin. To account for potential title changes or indexing differences, searches used combinations of author names, supervisors, and key terms, following established methodological standards^13,14^.

## RESULTS

### Thematic distribution and co-occurrence patterns

**Figure 1:**
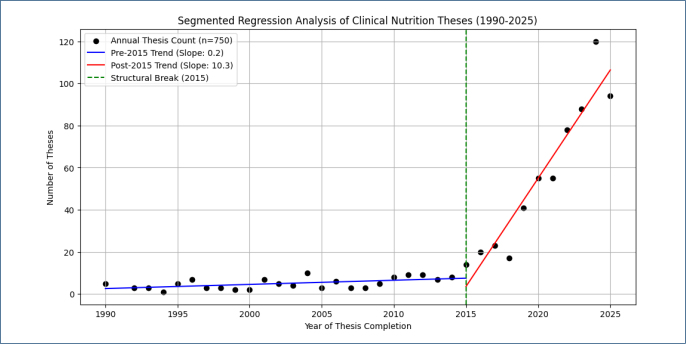
Segmented regression analysis of thesis production trends (1990–2025). Annual thesis production rate and the structural break point in 2015 (p<0.001). The graph shows the dramatic change in β coefficients and the growth momentum of the literature.

The thematic analysis of 750 specialist theses in the field of clinical nutrition demonstrated a clear concentration within specific research domains. The most frequently studied theme was sarcopenia and muscle health (36.5%, n=274), followed by nutritional support interventions (28.0%, n=210) and malnutrition screening or scoring systems (26.4%, n=198). The overall thematic distribution differed significantly from a uniform distribution model (χ^2^=412.5, p<0.001) (Figure 1).

Co-occurrence analysis revealed significant associations between sarcopenia and frailty (n=55, φ=0.51, p<0.001), as well as between sarcopenia and imaging-based assessment methods such as computed tomography and ultrasonography (n=32, φ=0.48, p<0.001). Malnutrition screening tools were frequently observed alongside both nutritional support-focused theses and sarcopenia-related studies, indicating substantial thematic overlap across these domains (Figure 1).

### Strategic thematic structure (Callon-based mapping)

**Figure 2:**
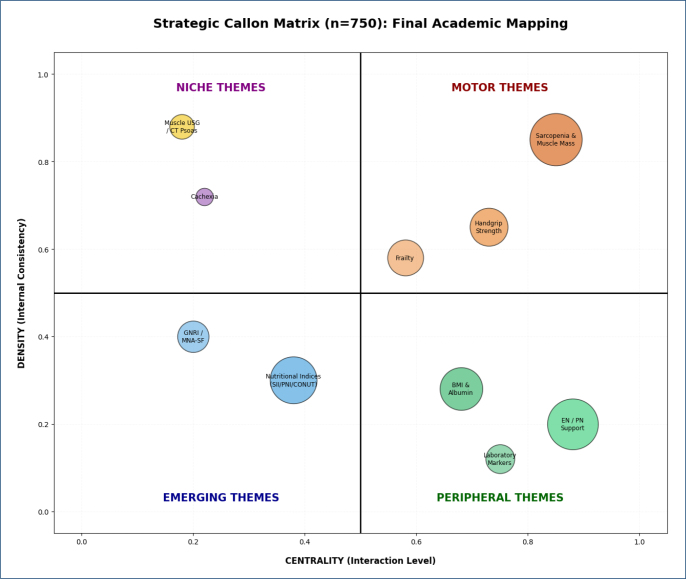
Thematic positioning of research topics in clinical nutrition based on Callon analysis.

Thematic mapping based on centrality and density metrics classified sarcopenia, muscle mass, and frailty as motor themes. Cachexia and imaging-based measurements, including psoas or muscle ultrasonography assessments, were identified as niche themes. Composite nutritional indices, such as the Systemic Immune-Inflammation Index (SII), Prognostic Nutritional Index (PNI), and Controlling Nutritional Status (CONUT) scores, were positioned within the emerging themes category (Figure 2).

### Specialty-specific thematic focus

A statistically significant association was observed between medical specialties and thematic focus (χ^2^=156.4, p<0.001). The detailed distribution, including adjusted residuals, is presented in Table 1. In pediatrics, malnutrition screening and nutritional risk assessment were thematically predominant, whereas sarcopenia and frailty were most prominent within geriatric theses. Enteral and parenteral nutrition interventions emerged as dominant themes in theses originating from general surgery and intensive care units (Figure 2).

**Table 1:** Specialty-based thematic distribution of clinical nutrition theses.

Medical specialty	Total theses (n)	Sarcopenia and muscle health (%)	Nutritional support (%)	Malnutrition screening (%)	Cachexia (%)	Adjusted residual (sarcopenia)
Geriatrics	201	70.6%	9.0%	15.9%	4.5%	**+6.1**
Pediatrics	156	11.5%	26.9%	55.1%	6.4%	-4.8
General surgery	144	16.7%	61.8%	19.4%	2.1%	-3.2
General internal medicine	127	40.9%	29.9%	25.2%	3.9%	+1.8
Intensive care	76	21.1%	64.5%	13.2%	1.3%	-2.1
Other specialties	58	37.9%	41.4%	17.2%	3.4%	–
Total	750	36.5%	28.0%	26.4%	1.2%	

Note: Pearson chi-square=156.4, p<0.001. Adjusted residuals >+2.0 indicate significantly higher representation than expected. Geriatrics shows a statistically significant concentration of sarcopenia-focused research. Bold values indicate statistically significant adjusted residuals (>+2.0 or <-2.0), highlighting categories with a substantially higher or lower representation than expected under the null hypothesis.

### Publication outcomes

**Figure 3:**
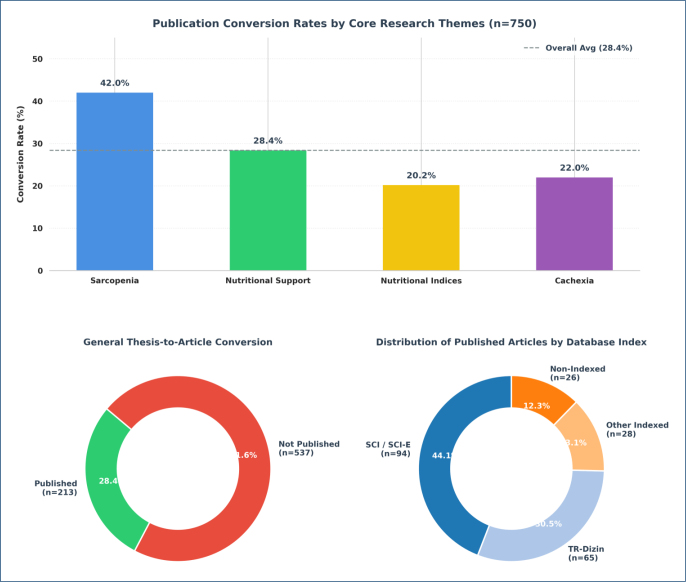
Peer-reviewed publication rates of theses according to primary thematic focus.

Of the 750 theses included in the analysis, 213 (28.4%) were published in peer-reviewed journals. Publication rates differed significantly according to thematic focus (χ^2^=32.4, p=0.018). The highest publication rate was observed in sarcopenia-focused theses (42.0%, 95%CI 36.2–47.8), whereas the lowest rate was recorded in cachexia-focused theses (22.2%, 95%CI 16.5–27.5). Among the published studies, 44.1% appeared in Science Citation Index/Science Citation Index Expanded (SCI/SCI-E) indexed journals and 30.5% in journals indexed in TR-Dizin (Figure 3).

### Temporal trends and structural change

Analysis of thesis production between 1990 and 2025 identified 2015 as a statistically significant structural breakpoint (Davies’ test, p<0.001). Prior to 2015, the annual growth rate in thesis production was β=0.42 (p=0.008), whereas after 2015 this rate increased to 2.84 theses per year (p<0.001). A level increase of β=12.4 (p<0.001) was observed at the breakpoint. The segmented regression model accounted for 88% of the total variance in thesis production over time (R^2^=0.88).

The statistical parameters of the segmented regression model, including annual growth rates (β coefficients), level changes, and 95%CI, are detailed in Table 2.

**Table 2:** Segmented regression analysis of thesis production trends in clinical nutrition (1990–2025).

Period/parameter	β coefficient	Standard error	p-value	95%CI
Pre-2015 period (1990–2015)	0.42	0.15	0.008	0.12–0.72
Post-2015 period (2016–2025)	2.84	0.41	<0.001	2.03–3.65
2015 breakpoint (level change)	12.4	2.18	<0.001	8.12–16.68
Model fit (R2)	**0.88**	–	–	–

Note: Segmented regression analysis was performed using Davies test to identify structural breaks. The breakpoint in 2015 was statistically significant (p<0.001), indicating a fundamental shift in thesis production momentum. The model explains 88% of the total variance in annual thesis production over the 35-year study period. CI: confidence interval. Bold values indicate statistically significant independent variables and model parameters (p<0.05).

## DISCUSSION

This bibliometric analysis indicates that medical specialty theses in clinical nutrition in Turkey have not only increased quantitatively over the past thirty-five years but appear to have undergone a significant conceptual and methodological transformation. The structural break identified after 2015 suggests that national academic production has shifted toward a muscle-focused phenotyping paradigm, showing notable alignment with broader global trends in clinical nutrition research^1,2^.

The overrepresentation of sarcopenia and frailty within geriatric theses appears to align closely with the strategic priorities emphasized by the European Society for Geriatric Medicine (EuGMS). Geriatric clinics seem to have played a central role in methodological advancement by incorporating “opportunistic imaging” approaches—specifically computed tomography (CT)-based muscle assessments derived from routine clinical scans. This strategy, identified in a notable proportion of the analyzed theses, suggests a potential shift toward a cost-effective and patient-safe model for body composition analysis and appears to be emerging as a prevalent methodological framework in national thesis research^15,16^.

The thematic shift observed in the Turkish academic landscape mirrors broader global trends identified in recent bibliometric inquiries. International literature similarly highlights a decisive transition from generalized nutritional markers toward specific muscle-centered phenotypes, a trajectory that gained significant momentum following the dissemination of the EWGSOP2 and GLIM criteria. However, a notable divergence emerges regarding complex metabolic wasting syndromes. While high-impact global research increasingly prioritizes cancer cachexia, our findings suggest that cachexia remains a relatively “under-developed” or peripheral theme within Turkey’s national academic output. This discrepancy indicates that while the Turkish research community has successfully aligned itself with the “muscle-focused” paradigm, there remains a critical need to integrate more complex wasting syndromes into the national research agenda to achieve full synchrony with evolving global clinical nutrition priorities.

The acceleration in thesis production observed after 2015 appears to coincide with key institutional and conceptual milestones in clinical nutrition practice in Türkiye. These include the widespread adoption of EWGSOP criteria and the formal recognition of sarcopenia as a disease entity through its inclusion in the International Classification of Diseases, Tenth Revision (ICD-10) classification (M62.84)^5^. Notably, the emphasis on muscle mass as a central component of nutritional assessment in Turkish academic output seems to have preceded the publication of the GLIM criteria in 2019^1^. The frequent use of GLIM-based approaches in later theses may reflect both this early conceptual alignment and the ongoing need for standardized diagnostic frameworks despite methodological differences between existing guidelines^1,2^. Furthermore, this structural breakpoint likely reflects the long-term impact of national educational initiatives, such as the “Life Long Learning” (LLL) modules and clinical nutrition congresses organized by national societies like Turkish Society of Clinical Enteral Parenteral Nutrition (Klinik Enteral Parenteral Nütrisyon Derneği), which systematically integrated nutritional assessment into medical residency curricula during this period.

The dominance of geriatrics in residual analyses appears to align extensive literature linking muscle loss to adverse outcomes and mortality in older adults^17,18^. Although the conceptual boundaries between sarcopenia and frailty remain debated^17^, the thematic patterns observed in this study indicate that national academic production may have gravitated toward a pragmatic, function-oriented perspective. The high proportion of muscle measurement–focused studies could be interpreted as the search for objective phenotyping tools. The emergence of muscle ultrasonography as a developing theme further suggests a growing academic interest in bedside, functional, and radiation-free assessment methods alongside conventional imaging techniques^19^.

Conversely, cachexia emerged as the least represented theme within the analyzed dataset. However, our analysis revealed a subtle exception in geriatric theses, where cachexia reached a relatively higher representation (4.5%) compared to the overall average (1.2%). This finding suggests that, while cachexia remains a peripheral theme nationally, geriatric medicine—likely driven by focus areas such as onco-geriatrics and advanced frailty management—exhibits a stronger academic interest in this complex wasting syndrome. Nevertheless, this level of focus is still insufficient when compared to international priorities, particularly within domains featured as central themes of the 2025 EuGMS Reykjavik Congress^20^. The paucity of academic focus on cachexia may be linked to its clinical perception as a refractory or irreversible end-stage condition, or it may reflect insufficient multidisciplinary collaboration between oncology and geriatric medicine^21,22^. Nevertheless, in consideration of the systemic inflammatory nature of cachexia and its robust association with adverse clinical outcomes, the findings of this study suggest that earlier and more integrated research efforts may be strategically essential to bridge this national academic gap^23,24^.

The temporary slowing of thesis production during the COVID-19 pandemic may potentially reflect restrictions on clinical research activities. Nevertheless, increased awareness of the relationship between muscle loss, immunity, and clinical resilience during this period could have acted as a catalyst the continuity of academic interest in the post-pandemic era^22^.

The overall publication conversion rate of 28.4% appears broadly consistent with previously reported rates for medical specialty theses in Turkey^14^. The higher publication success observed in sarcopenia-focused theses (42.0%) may be partially attributed to the objective measurability of outcomes and sustained international editorial interest in this field. Despite the dearth of comparative bibliometric data on thematic publication rates from other countries, the preponderance of sarcopenia in high-impact journals is indicative of the global shift toward phenotype-based clinical nutrition research. This finding indicates that the selection of sarcopenia as a research theme in Turkey holds considerable potential for facilitating international publication, a phenomenon that stands in contrast to the predominance of more conventional nutritional subjects in this field. Furthermore, discrepancies with higher publication rates reported in specialist geriatric studies could be interpreted as a reflection of the broader thematic diversity and the substantially larger sample size analyzed in the present study^25^.

The findings of this study offer critical implications for clinical practice. The evident shift toward a “muscle-centered” paradigm reinforces the need to integrate international phenotyping criteria, such as GLIM and EWGSOP2, into routine clinical workflows. Our results advocate for the broader adoption of “opportunistic imaging” (CT-based muscle assessment) across non-geriatric specialties, such as oncology and surgery, as a cost-effective diagnostic tool that requires no additional radiation. Furthermore, the significant academic void identified in the field of cachexia underscores the urgent necessity for developing robust multidisciplinary protocols and targeted physician training to address this complex syndrome more effectively.

A primary strength of this study lies in its position as the first and most comprehensive national mapping of clinical nutrition literature in Turkey, spanning a substantial 35-year period through the lens of medical specialty theses. Beyond simple descriptive statistics, the implementation of advanced bibliometric techniques—such as Callon’s centrality and density metrics and segmented regression models—provides a mathematically robust validation of thematic evolution. Furthermore, the manual screening of 750 theses and the high inter-rater reliability achieved during the process ensure the representativeness and scientific integrity of the analyzed dataset.

### Limitations

This study is limited to medical specialty and subspecialty theses and does not include contributions from other health disciplines, such as nutrition and dietetics, which may restrict the representation of the full multidisciplinary landscape of the field. Furthermore, as the analysis relied on a national database with inherent indexing heterogeneity, minor interpretive biases or coding discrepancies cannot be entirely excluded despite high inter-rater reliability. The assessment of thesis-to-publication conversion was conducted through selected international and national databases, meaning some published outputs indexed elsewhere may have been missed. Finally, as a bibliometric study, these findings primarily reflect academic research trends and thematic evolutions rather than the direct clinical quality of the included studies.

## CONCLUSION

In conclusion, this study demonstrates that clinical nutrition research in Turkey has evolved over the past 35 years from a general focus on malnutrition toward a methodologically mature, muscle-centered, and phenotype-based paradigm. Geriatric medicine has served as the primary driver of this transformation, with the integration of “opportunistic imaging” approaches significantly enhancing the quality of academic output. While sarcopenia has moved to the center of national research, complex wasting syndromes such as cachexia remain academically neglected. Although this evolution has improved international alignment and academic visibility, these findings underscore the urgent need for future research agendas to focus on clinically critical yet underrepresented areas like cachexia through more holistic and multidisciplinary strategies.

## Data Availability

The datasets generated and/or analyzed during the current study are available from the corresponding author upon reasonable request.

## References

[1] Cederholm T, Jensen GL, Correia MITD, Gonzalez MC, Fukushima R, Higashiguchi T (2019). GLIM criteria for the diagnosis of malnutrition - a consensus report from the global clinical nutrition community.. J Cachexia Sarcopenia Muscle.

[2] Schuetz P, Seres D, Lobo DN, Gomes F, Kaegi-Braun N, Stanga Z (2021). Management of disease-related malnutrition for patients being treated in hospital.. Lancet.

[3] Norman K, Pichard C, Lochs H, Pirlich M (2008). Prognostic impact of disease-related malnutrition.. Clin Nutr.

[4] Schueren MAE, Keller H, Cederholm T, Baracos V, Mueller C, Jensen GL (2018). Malnutrition screening tools: does one size fit all? A systematic review.. Proc Nutr Soc.

[5] Cruz-Jentoft AJ, Bahat G, Bauer J, Boirie Y, Cederholm T (2019). Sarcopenia: revised European consensus on definition and diagnosis.. Age Ageing.

[6] Sarbay I, Turna A (2022). Thoracic surgery in Turkey.. J Thorac Dis.

[7] Uzun H, Ergin AB (2019). Publication rates of Turkish medical specialty theses in surgery.. J Surg Res.

[8] Kılıçgün K, Tanju S, Toker A (2023). Bibliometric spotlight on thoracic surgery specialization theses in Turkey.. Ann Thorac Cardiovasc Surg.

[9] Güneş M, Kocaaslan R, Ceccarelli G, Şahin S (2018). Publication rates of dissertations in urology: a Turkish national database analysis.. Turk J Urol.

[10] Donthu N, Kumar S, Mukherjee D, Pandey N, Lim WM (2021). How to conduct a bibliometric analysis: an overview and guidelines.. J Bus Res.

[11] Callon M, Courtial JP, Laville F (1991). Co-word analysis as a tool for describing the network of interactions between basic and technological research: the case of polymer chemistry.. Scientometrics.

[12] IBM Corp (2019). IBM SPSS Statistics for Windows, Version 26.0..

[13] Zimmermann R, Fröhlich S, Behringer W, Gareis R, Handler C, Heinz P (2019). Interventions to increase research publications in graduate medical education trainees.. Arch Med Sci.

[14] Özdemir M, Abuşoğlu S, Ünlü A (2024). A bibliometric analysis of biochemistry theses in Türkiye: comparison with global trends.. Turk J Biochem.

[15] Mourtzakis M, Prado CM, Lieffers JR, Reiman T, McCargar LJ, Baracos VE (2008). A practical and precise approach to quantification of body composition in cancer patients using computed tomography images acquired during routine care.. Appl Physiol Nutr Metab.

[16] Prado CM, Heymsfield SB (2014). Lean tissue imaging: a new era for nutritional assessment and intervention.. JPEN J Parenter Enteral Nutr.

[17] Clegg A, Young J, Iliffe S, Rikkert MO, Rockwood K (2013). Frailty in elderly people.. Lancet.

[18] Vellas B, Fielding RA, Bens C, Bernabei R, Cawthon PM, Cederholm T (2018). Implications of ICD-10 for sarcopenia clinical practice and clinical trials: report by the ınternational conference on frailty and sarcopenia research task force.. J Frailty Aging.

[19] Perkisas S, Bastijns S, Baudry S, Bauer J, Beaudart C, Beckwée D (2021). Application of ultrasound for muscle assessment in sarcopenia: 2020 SARCUS update.. Eur Geriatr Med.

[20] European Geriatric Medicine Society. (2025). 21st EuGMS Congress: geriatric medicine in a changing world.. Reykjavik.

[21] Muscaritoli M, Anker SD, Argilés J, Aversa Z, Bauer JM, Biolo G (2010). Consensus definition of sarcopenia, cachexia and pre-cachexia: joint document elaborated by Special Interest Groups (SIG) “cachexia-anorexia in chronic wasting diseases” and “nutrition in geriatrics”.. Clin Nutr.

[22] Fearon K, Strasser F, Anker SD, Bosaeus I, Bruera E, Fainsinger RL (2011). Definition and classification of cancer cachexia: an international consensus.. Lancet Oncol.

[23] Baracos VE, Martin L, Korc M, Guttridge DC, Fearon KCH (2018). Cancer-associated cachexia.. Nat Rev Dis Primers.

[24] Arends J, Strasser F, Gonella S, Solheim TS, Madeddu C, Ravasco P (2021). Cancer cachexia in adult patients: ESMO Clinical Practice Guidelines*.. ESMO Open.

[25] Tokatlı M, Balcı C (2025). Academic outcomes of geriatric medicine theses in internal medicine residency: a 25-year nationwide review in Türkiye.. BMC Med Educ.

